# Quantitative structural analysis of hemifacial microsomia mandibles in different age groups

**DOI:** 10.3389/fped.2023.1157607

**Published:** 2023-04-17

**Authors:** Ziwei Zhang, Xiaojun Chen, Byeong Seop Kim, Wenqing Han, Yingjie Yan, Xuetong Wang, Xin Li, Yan Zhang, Gang Chai

**Affiliations:** Department of Plastic and Reconstructive Surgery, Shanghai Ninth People’s Hospital, Shanghai JiaoTong University School of Medicine, Shanghai, China

**Keywords:** hemifacial microsomia, mandibular asymmetry, mandibular body, mandibular ramus, progression

## Abstract

**Introduction:**

This study aims to quantitively analyze mandibular ramus and body deformities, assessing the asymmetry and progression in different components.

**Methods:**

This is a retrospective study on hemifacial microsomia children. They were divided into mild/severe groups by Pruzansky-Kaban classification and into three age groups (<1 year,1–5 years, 6–12 years old). Linear and volumetric measurements of the ramus and the body were collected via their preoperative imaging data to compare between the different sides and severities, using independent and paired tests, respectively. The progression of asymmetry was assessed by changes in affected/contralateral ratios with age using multi-group comparisons.

**Results:**

Two hundred and ten unilateral cases were studied. Generally, the affected ramus and body were significantly smaller than those on the contralateral side. Linear measurements on the affected side were shorter in the severe group. Regarding affected/contralateral ratios, the body was less affected than the ramus. Progressively decreased affected/contralateral ratios of body length, dentate segment volume, and hemimandible volume were found.

**Discussion:**

There were asymmetries in mandibular ramus and body regions, which involved the ramus more. A significant contribution to progressive asymmetry from the body suggests treatment focus in this region.

## 1. Introduction

Hemifacial microsomia (HFM) is a congenital craniofacial deformity, with a prevalence of 1/5,600–1/3,000 of live births (1). HFM mainly involves the structures originating from the first and second branchial arches, resulting in the hypoplasia of the craniofacial skeleton and surrounding soft tissues, including the maxilla, mandible and zygoma, on one or both sides (2). It may also involve various extracranial systems such as circulatory, respiratory, genitourinary and skeletal systems (3). Among these skeletal malformations, mandibular dysplasia is the cornerstone with a complex presentation (4). Both the mandibular ramus and the body can be dysplastic, causing asymmetry in the lower face, bringing great aesthetic and functional influences and a significant psychological burden to the patients and their families (4).

The widespread method used to evaluate mandibular deformities is the Pruzansky–Kaban classification, which is based primarily on ramus/condyle deficiency and temporomandibular joint function (5). This classification system is clinically significant for diagnosis and treatment guidance; however, it does not address morphological analysis of the mandibular ramus or body (4). Particularly, quantitative studies on the affected mandibular body in hemifacial microsomia are inconclusive (6–11). Some studies considered it smaller than the contralateral side, causing clinical difference; while compensatory body growth was also found, with an incidence of up to 14.1% in the study population (6–10).

Mandibular distraction osteogenesis (MDO) is the primitive surgical treatment for correcting skeletal asymmetries in hemifacial microsomia. Preoperative planning of distraction, including osteotomy sites and distraction length, is directly related to postoperative outcome (12). Besides, the procedure timing remains controversial. Clinical concerns include psychosocial problems, asymmetry progression, and possible recurrence requiring secondary intervention (13). Some studies suggested that mandibular deformities progress with age and that early intervention is beneficial to avoid further deformities (14–16). Still, the time point for MDO during development remains unclear (5, 16–20). Thus, a comprehensive understanding of the mandibular deformity's morphological characteristics and the developmental pattern is vital for a preoperative plan.

We aime to assess mandibular ramus and body asymmetries at different severity levels using preoperative three-dimensional computed tomographic (3D-CT) data of growing patients with hemifacial microsomia, and to demonstrate the growth pattern in different regions by comparisons among different age groups. This study aims to provide clues to the pathogenesis of mandibular dysplasia in hemifacial microsomia and assist in clinical decision-making regarding distraction osteogenesis.

## 2. Materials and methods

### 2.1. Subjects

This was a retrospective study that collected 3D-CT data from consecutive patients up to 12 years of age with hemifacial microsomia at our institution from 2014 to 2021. Patients were diagnosed by a senior craniomaxillofacial surgeon through clinical presentation and CT images and were assessed by Pruzansky–Kaban classification. Type I and IIa were categorized as the mild group, while type IIb and III were the severe group. We selected patients with unilateral involvement for inclusion in the study and excluded patients with other craniomaxillofacial deformities or trauma. The included patients were divided into different age groups, <1 year, 1–5 years, and 6–12 years, according to the altered developmental pattern of the mandible at different stages of growth (21). Informed consent was obtained from patients to obtain imaging data, and the study was approved by the ethics committee (SH9HIEC-2018-159-T117).

### 2.2. Three-dimensional reconstruction and measurements

**Figure 1:**
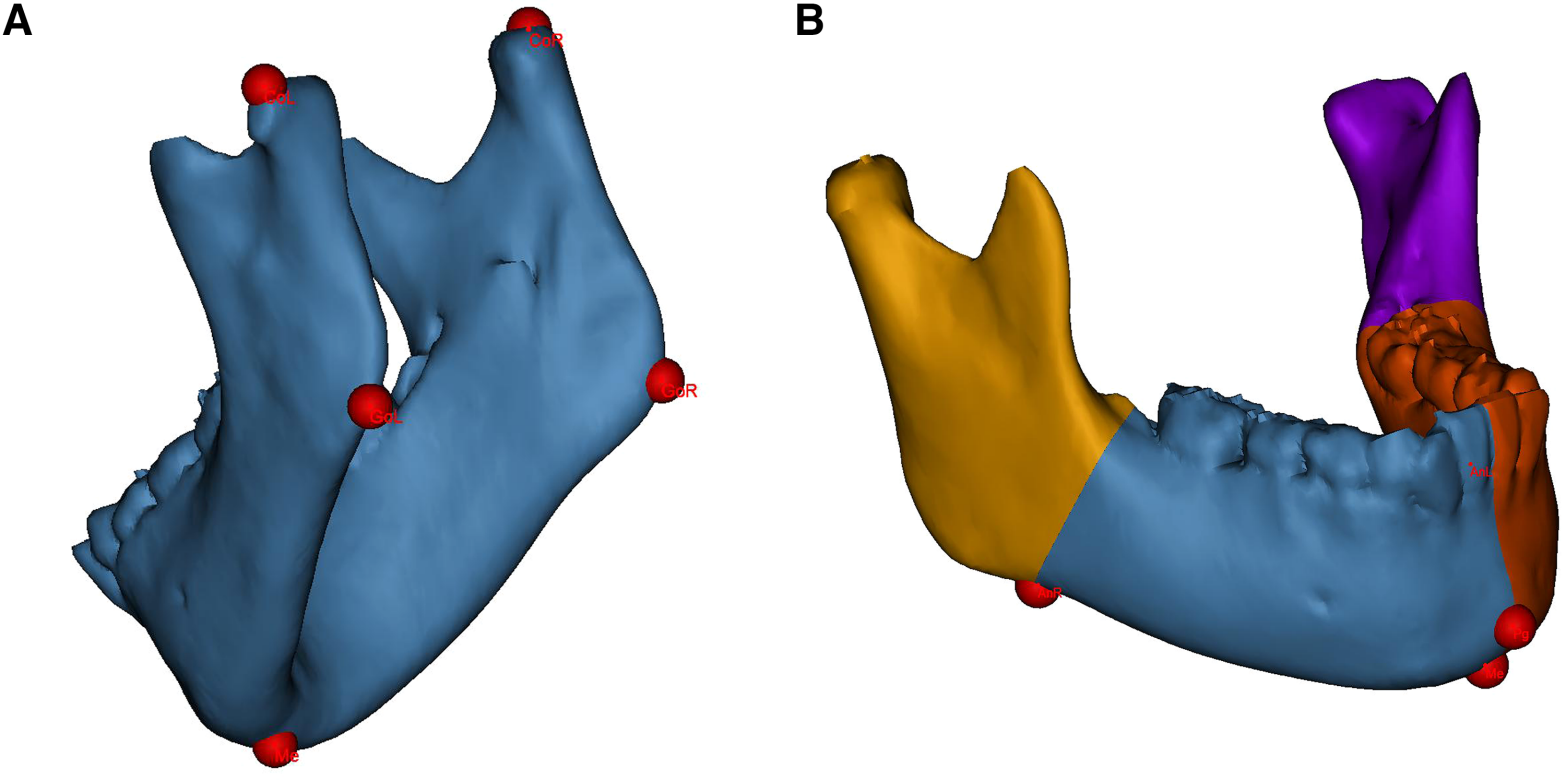
Landmarks identification and mandibular segmentation on the 3D model. (**A**) landmarks for linear measurements. (**B**) The mandible is divided into the proximal segments and the dentate segments for volumetric measurements. CoL, condylion left; CoR, condylion right; GoL, gonion left; GoR, gonion right; Me, menton; Pg, pogonion; AnL, antegonial notch left; AnR, antegonial notch right.

All measurements were performed by unknowing third-party researchers. The original 3D-CT data (Brilliance 64 CT scanner, Philips, the Netherlands) were imported into the medical image processing software Mimics 21.0 (Materialise, Leuven, Belgium) in Digital Imaging and Communications in Medicine (DICOM) format. The mandible was segmented and 3D-reconstructed. Landmarks were identified on the 3D mandibular model, as shown in Figure 1A.

Linear measurements included the mandibular ramus height and body length on the affected and contralateral sides. The ramus height was defined as the distance from the condylion to the ipsilateral gonion; the body length was defined as the distance from the gonion to the menton (Me) (22). Volumetric measurements included the hemimandible volume as well as the bilateral proximal segment and dentate segment volumes. The hemimandible is divided according to the central incisor embrasure, Me and pogonion (Pg); the dividing divisions of the proximal and dentate segments consist of the antegonial notch and the developing second molar follicle (Figure 1B) (6).

The affected side values to the contralateral side values (A/C ratio) of linear and volumetric measurements were also calculated. Because of varying degrees of structural deficiencies, the landmarks on the affected side were approximately identified. The uppermost distal point and lowermost distal point were chosed, which show anatomical similarity to condylion and gonion, respectively, of the contralateral side (23).

### 2.3. Statistical analysis

To assess the reliability of the measurements, inter-examiner and intra-examiner reliability were analyzed. Two independent third-party researchers performed the same measurements, respectively. Repeated measurements were performed by the same researchers at an interval of two weeks. The inter-researcher and the intra-researcher reliability was assessed by the intra-class correlation coefficient (ICC). ICCs of 0.81–1.00 indicate almost perfect agreement (24). The mean values were finally calculated for further analysis.

Normality was tested by the Shapiro–Wilk test and homogeneity of variance by the Levene test for all quantitative data. If the data showed normality and homogeneity of variance, independent *t*-tests were used to compare the differences in measurements by gender, sidedness, and severity; paired *t*-tests were used to compare the differences between the contralateral and affected sides in the same mandibular model; ANOVA tests were used to compare the differences between different age groups. Otherwise, nonparametric tests were used.

*P* < 0.05 was considered a statistical difference. All statistical analyses were completed by SPSS 26.0 (IBM Corp., Armonk, NY, USA).

## 3. Results

### 3.1. Patients' characteristics

A total of 210 mandibular models were finally analyzed. The mean age of the patients was 3.73 years (2 months-11 years old). 60.5% of them were boys, while there was no obvious predominance in the sidedness (left: 50%, right: 50%). Nearly half of the patients (48.1%) manifested mild mandibular dysplasia. Their clinical data are shown in Table 1.

**Table 1:** Demographic information and baseline characteristics.

	Mild group	Severe group	Subtotal
**Gender**			
Male	62	55	127
Female	39	44	83
**Sidedness**			
Left	52	53	105
Right	49	56	105
**Age group**			
<1 year old	22	36	58
15 years old	43	46	89
6–12 years old	36	27	63
Total	101	109	210

### 3.2. Inter-examiner and intra-examiner reliability

The ICC calculation results are shown in Table 2. The intra-researcher reliability was assessed for both of them. Each one's mean values were used for inter-researcher reliability analysis. Intra- (ICC 0.946 −0.995 for Researcher 1 and ICC 0.952–0.991 for Researcher 2) and inter-researcher reliability (ICC 0.958–0.993) were both high. All measurements showed good reliability.

**Table 2:** Inter- and intra-researcher reliability analyses.

	ICC	95% CI
**Intra-researcher reliability**		
Researcher 1		
Ramus height (mm)	0.988	0.952–0.997
Body length (mm)	0.995	0.980–0.999
Hemimandibular volume (mm^3^)	0.971	0.890–0.993
Proximal segment volume (mm^3^)	0.946	0.800–0.986
Dentate segment volume (mm^3^)	0.983	0.934–0.996
Researcher 2		
Ramus height (mm)	0.984	0.938–0.996
Body length (mm)	0.991	0.966–0.998
Hemimandibular volume (mm^3^)	0.959	0.845–0.990
Proximal segment volume (mm^3^)	0.952	0.821–0.988
Dentate segment volume (mm^3^)	0.975	0.902–0.994
**Inter-researcher reliability**		
Ramus height (mm)	0.988	0.952–0.997
Body length (mm)	0.993	0.972–0.998
Hemimandibular volume (mm^3^)	0.964	0.861–0.991
Proximal segment volume (mm^3^)	0.958	0.831–0.990
Dentate segment volume (mm^3^)	0.979	0.917–0.995

ICC, intra-class correlation; CI, confidence interval.

### 3.3. Linear and volume measurements

There was no significant effect of gender or sidedness on the mandibular measurements. All measurements were observed with a significant increase corresponding to increasing age (*P* < 0.001).

#### 3.3.1. Comparison between the affected/contralateral sides

**Figure 2:**
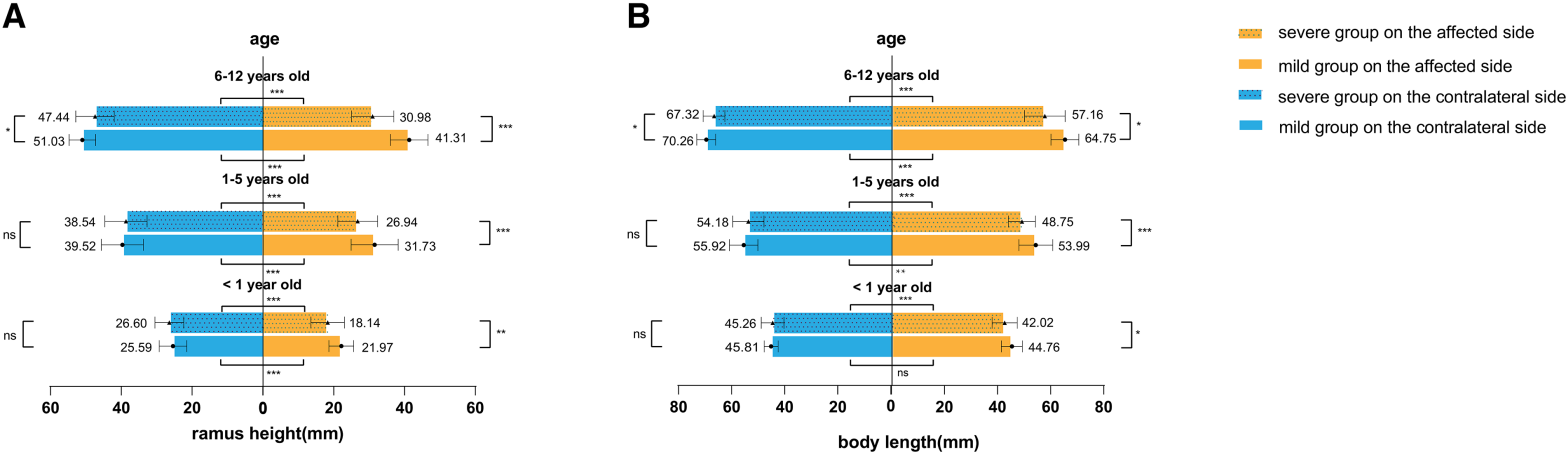
Linear analysis. (**A**) Ramus height and (**B**) body length were compared between different severities and sides. *P < 0.05, **P < 0.01, ***P < 0.001, ns, no significance.

**Figure 3:**
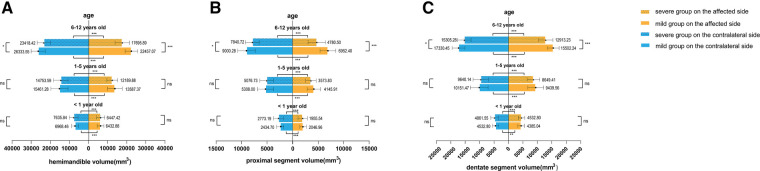
Volumetric analysis. (**A**) Hemimandible volume, (**B**) proximal segment volume, and (**C**) dentate segment volume were compared between different severities and sides. *P < 0.05, **P < 0.01, ***P < 0.001, ns, no significance.

In different age groups, the ramus height, body length, hemimandible volume, proximal segment volume, and dentate segment volume were smaller on the affected side than on the contralateral side in both the mild and severe groups (Figures 2, 3), except for body length on the affected side in mild patients younger than one year of age (*t* = 1.980, *P* = 0.061).

#### 3.3.2. Comparison between the mild/severe groups

In addition, we found that in patients younger than six years old, only the linear measurements of the affected side differed between mild and severe groups (younger than 1 year old: 21.97 vs. 18.14 mm in ramus height and 42.02 vs. 44.76 mm in body length; 1–5 years old: 26.94 vs. 31.73 mm in ramus height and 48.75 vs. 53.99 mm in body length). Meanwhile, there were no significant differences in the contralateral mandible or the volumetric measurements of the affected side. However, in the age group of 6–11 years old, significant differences were observed in all measurements, including on the relatively less affected side of mandibles set as controls in this study. The severe group exhibited a “smaller” contralateral hemimandible (Figures 2, 3).

## 4. Affected/contralateral ratio

### 4.1. The asymmetries in different regions

We assessed the mandibular asymmetry by the A/C ratio, which was generally smaller in the severe group, i.e., the asymmetry of the mandibular ramus and body were more significant in linear and volumetric measurements (Table 3). By observing the A/C ratio, it was found that the values of the body A/C ratios, including linear and volumetric asymmetry, were smaller than those of the ramus.

**Table 3:** Comparisons of A/C ratio in different age groups.

	Ramus height			Body length			Hemi-mandibular volume			Proximal segment volume			Dentate segment volume		
	Mild group	Severe group	*P*	Mild group	Severe group	*P*	Mild group	Severe group	*P*	Mild group	Severe group	*P*	Mild group	Severe group	*P*
<1 year old	0.86 ± 0.06	0.68 ± 0.13	<0.001*	0.98 ± 0.06	0.93 ± 0.09	0.023*	0.92 ± 0.05	0.84 ± 0.08	<0.001	0.84 ± 0.09	0.69 ± 0.16	0.001*	0.97 ± 0.05	0.92 ± 0.08	0.002*
1–5 years old	0.80 ± 0.13	0.70 ± 0.13	<0.001	0.97 ± 0.07	0.90 ± 0.08	<0.001*	0.89 ± 0.08	0.84 ± 0.07	0.001	0.79 ± 0.13	0.72 ± 0.17	0.029	0.94 ± 0.07	0.91 ± 0.07	0.009*
6–12 years old	0.82 ± 0.10	0.65 ± 0.11	<0.001	0.92 ± 0.06	0.87 ± 0.09	0.030*	0.86 ± 0.06	0.77 ± 0.09	<0.001	0.78 ± 0.12	0.63 ± 0.18	0.001*	0.91 ± 0.07	0.85 ± 0.07	0.003
*P*	0.14	0.201		0.001^†^	0.020		0.007	0.002		0.163	0.206^†^		0.006^†^	<0.001^†^	

* Mann–Whitney test.

^†^ Kruskal–Wallis test.

### 4.2. The asymmetry progression with age

The A/C ratio of ramus length and proximal segment volume did not change significantly with age, regardless of whether the mandibular deformity was mild or severe. On the other hand, body length, hemimandible volume and dentate segment volume showed significant differences, with a tendency for the A/C ratio to decrease in older age groups (Table 3), suggesting that the asymmetry of the body increased with age and contributed to the overall asymmetry of the mandible.

## 5. Discussion

This study's quantitative analysis of mandibular ramus and body showed their evident asymmetries in HFM, which engaged the ramus more and were more serious in type IIB/III abnormalities. In the severe group, mandibular hypoplasia spread to the contralateral side. The results also revealed the considerable contribution of the body to the progressive asymmetry.

The findings of a significant reduction in body size on the affected side are generally consistent with the previous studies (6–8, 10). We further found that the degree of asymmetry of the body was generally smaller than that of the ipsilateral ramus, and no significant shortening of the body was observed in infancy. This may be because they act as relatively independent regions with different primordium and have distinctive osteogenic patterns during embryonic and postnatal development (25). The proximal segment roughly includes the ramus and condylar regions and is predominantly endochondral osteogenic; the dentate segment is intramembranous osteogenic. Kim et al. revealed that HFM involves the mandibular body unit least and the condylar unit to the greatest extent, suggesting that the main onset of HFM is in the anatomical vicinity of the condyle development (8). Of course, the findings of morphological analysis could only serve as a hint, and exploring the underlying mechanisms requires intensive research.

In patients at a later stage of development, the volumetric data on the affected side, even together with the contralateral side, also showed significant differences between severity levels. The growth potential is diminished in the severe group with rather severe ramus/condyle malformation. The deficiency at birth may lead to an adaptive structural remodeling, even affecting the contralateral side, as observed in this study. Similarly, volumetric analysis from Steinbacher et al. showed that the proximal and dental-bearing segment decreased in volume with increasing Pruzansky score (6). Steinbacher et al. and Kaya et al. also discovered that the contralateral mandible was reduced in severe patients compared to controls without HFM, which suggested a bilateral nature of the cases (6, 22). However, none of them observed differences in the changes between ages in young children. It is noted that patients diagnosed with unilateral HFM often have relatively minor skeletal or soft tissue abnormalities on the contralateral side (26).

Regarding the mandibular progression, some studies concluded progressive distortion of the facial bones on the affected and contralateral sides (14–16). Others argued that the asymmetry was not progressive and surgical outcomes would be compromised by recurrence (27–30). Our analysis of a large sample verified progression in both the mild and severe groups. However, the data supporting it came from body-related measurements, not the ramus region. The progressive body asymmetry with age could result from certain functional factors, such as malocclusion disorders and muscle defects (8, 31). Our results indicate early intervention to prevent further deformities (16). Concerns about post-operative recurrence can be alleviated by overcorrection. Weichman et al. found higher long-term aesthetic satisfaction in patients with younger initial intervention age and greater overcorrection (20). A choice for early intervention might be before 5–6, when the two major parts of the mandible become distinctive in their growth pattern to accommodate mid-facial development (21).

In summary, besides the ramus, the body asymmetry significantly effected the whole mandible. Surgical focus on the body asymmetry besides ramal lengthening was also suggested. The oblique distraction vector needs to be considered to increase the anteroposterior dimension of the body (32). Quantitative preoperative imaging analysis could be used not only to describe morphology, but also help decide how much the distraction should be. Additionally, the surgeons need to consider the change in the growth potential and the postoperative bone retraction before making a comprehensive judgment (29, 33). Novel built-in distractors that enable bidirectional distraction might be an effective therapeutic tool. However, its application needs a series of model experiments and animal trials for feasibility verification.

There are some limitations in this study. We only performed two linear and three volumetric measurements on the ramus and the body, which provided limited information. More detailed morphological changes may be possible by constructing holistic geometric morphometrics of the mandible and performing a comprehensive analysis. In addition, complementary analysis of other adjacent structural factors, such as the tongue which have an important influence on mandibular morphology with a similar origin to the mandible, or any neuromuscular involvement might provide deeper insight into the pathogenesis of mandibular deformities (21, 34). Additionally, the present study is a cross-sectional study, which can only indicate the average for each age group. More longitudinal data are needed to assess detailed growth patterns of the mandibular components at the individual level. More ideally, including age-matched controls from a healthy population would help clarify the bilateral involvement.

In this study, we performed quantitative morphological analysis to assess the mandibular asymmetries of ramus and body in HFM. We found asymmetries in the mandibular ramus and body regions, with the ramus being more involved. A significant contribution to progressive asymmetry from the body, advising therapeutic concentrates on both mandibular regions for improved surgical outcomes.

## Data Availability

The original contributions presented in the study are included in the article/Supplementary Material, further inquiries can be directed to the corresponding author.
